# Early p38 Activation Regulated by MKP-1 Is Determinant for High Levels of IL-10 Expression Through TLR2 Activation

**DOI:** 10.3389/fimmu.2021.660065

**Published:** 2021-06-21

**Authors:** Sara Francisco, Alicia Arranz, Javier Merino, Carmen Punzón, Rosario Perona, Manuel Fresno

**Affiliations:** ^1^ DIOMUNE S. L., Parque Científico de Madrid, Madrid, Spain; ^2^ Centro de Biología Molecular Severo Ochoa, Universidad Autónoma de Madrid, Madrid, Spain; ^3^ Centro de Biología Molecular “Severo Ochoa”, Consejo Superior de Investigaciones Cientificas (CSIC)-Universidad Autónoma de Madrid, Madrid, Spain; ^4^ Instituto de Investigaciones Biomedicas, Alberto Sols Universidad Autónoma de Madrid, Madrid, Spain

**Keywords:** macrophages, innate immunity, toll-like receptors, MAPK signaling, cytokines

## Abstract

Toll-like receptors (TLRs) play a crucial role in the recognition of pathogen-derived components as a first line of defense against infections. It has been suggested that depending on the nature of the pathogens, TLRs activation induce a distinct cytokine profile that may contribute to the polarization of the acquired immune response. Here, we investigated the early MAPK signaling activation *via* TLR4 and TLR2 receptors and its impact in differential cytokine profile by macrophages. We found that TLR2 ligands activated MAPKs p38 and ERK earlier compared to the TLR4 ligand LPS in macrophages. Higher IL-10/IL-12 and IL-10/TNF-α ratios were also observed at later time points in response to TLR2 ligands compared to LPS. The results also indicate an earlier activation of the phosphatase MKP-1 and that MKP-1 KO macrophages show a prolongation in p38 phosphorylation in response to TLR2 stimulation. Furthermore, p38 is critical for IL-10 expression in response to TLR2 ligands, which triggers the macrophage change to a M2 and regulatory phenotype in contrast to the M1 phenotype induced by TLR4 activation. Therefore, the early TLR2-mediated p38 induction contributes for the high IL-10 production, likely as a virulence strategy to suppress host Th1 response against certain types of pathogens.

## Introduction

Innate immune mechanisms that follow recognition of microbe or danger signals influence the nature and magnitude of subsequent adaptive immune responses (1). Toll-like receptors (TLRs) are key components of the immune system with unique capacity to sense the presence of pathogens through binding to a vast array of conserved microbial components from bacterial, viral and fungal origin named pathogen-associated molecular patterns (PAMPs). This recognition triggers the activation of intracellular signaling pathways determinant to mount a rapid and effective immune response (2, 3).

Surface TLRs, especially TLR2 and TLR4 have gained importance due to their ability of recognize a diversified array of pathogenic ligands. TLR4 is known to complex with MD-2 and recognize the lipopolysaccharide LPS molecule, present in the gram-negative bacteria cell wall (4). TLR2 recognizes a wide array of ligands as lipopeptides, lipoteichoic acid from Gram-positive bacteria, lipoarabinomannan from mycobacteria, zymosan from fungi, tGPI-mucin from *Trypanosoma cruzi* and the hemagglutinin protein from measles virus (4). This receptor forms an m-shaped heterodimer with either TLR1 or TLR6 or recognition of tri- or di acylated lipopeptides respectively (5, 6).

Upon binding of microbial products, TLRs trigger a cascade of signals that induce the production of several cytokines determinant for pathogen killing and activation of the adaptive immunity system. Several previous studies have demonstrated that signaling by distinct TLRs is not fully equivalent leading to differential cytokine gene induction (7). Indeed, TLR4 agonists induced mostly proinflammatory cytokines such as TNF, IL-12 and IFN-β in contrast to TLR2 ligands, which induced preferentially IL-10, IL-8 and IL-23 in human DCs (8).

The activity of the MAPKs ERK and p38 are determinant in eliciting inflammatory responses through activation of multiple transcription factors and through the stabilization and translation of cytokines mRNA that contain AU-rich elements (9). MAP kinases are primarily inactivated by a group of dual-specificity protein phosphatases through dephosphorylation of the critical tyrosine and threonine residues of activated MAP kinases. Therefore, these phosphatases may serve as crucial feedback control regulators in the innate immune response during microbial infection. The phosphatase MKP-1 is highly expressed in macrophages and studies with MKP-1 KO peritoneal macrophages show that this phosphatase regulates MAPKs p38 and JNK with little effect on ERK (10, 11). Moreover, recent studies have demonstrated that p38 is determinant for IL-10 expression in response to LPS (12, 13).

Considering the previous studies that demonstrate a differential cytokine profile between TLR2 and TLR4 activation, we prompted to investigate whether this differential cytokine pattern can be ascribed to differences on MAPKs signaling pathways activation by TLR4 and TLR2 ligands. In our study, we demonstrate that the TLR2 ligands Pam3CSK4 (TLR2/1) and FSL-1 (TLR2/6) induce higher levels of anti-inflammatory IL-10 and lower levels of pro-inflammatory cytokines IL-12 and TNF-α compared to TLR4 ligands in mouse macrophages. TLR2 ligands activate MAPKs p38 and ERK earlier compared to TLR4 ligands and MKP-1 controls p38 phosphorylation in response to TLR2 activation. These results suggest that early p38 activation is critical for the higher levels of IL-10 induction in response to TLR2 ligands.

## Materials and Methods

### Reagents

TLR4 ligand LPS from E. coli O111:B4 (Sigma), TLR2/TLR1 ligand Pam3CSK4 and TLR2/6 ligand FSL-1 (InvivoGen) were resuspended in sterile PBS 1x. The inhibitors SB203580 (Cayman), 5Z-7-oxozeaenol (Calbiochem) (Merck) and FR180204 (Merck) were resuspended in DMSO and diluted in cell culture media achieving a final DMSO concentration of 0,1% (v/v).

### Cell Lines

The murine macrophage cell line RAW264.7 was cultured in RPMI 1640 medium (Gibco) (2 mM L-glutamine, antibiotics: 100 units/mL penicillin, 100 μg/mL streptomycin), supplemented with 5% FBS (Merck). Cells were cultured in 12-well plates at a density of 0,5x10^6^ cells/well and serum deprived for 16 h prior ligand stimulation.

### Isolation of Mouse Peritoneal Macrophages

C57BL/6 WT, TLR2 and TLR4 KO mice were obtained from S. Akira and MKP-1 KO mice from Bristol-Myers Squibb. All mice were bred and maintained in the animal facilities of the Centro de Biologia Molecular Severo Ochoa in Universidad Autonoma de Madrid. All animal procedures were performed in strict accordance with the European Commission legislation for the protection of animal used purposes (2010/63/EU). The protocol for the treatment of the animals was approved by the Comité de Ética de la Dirección General del Medio Ambiente de la Comunidad de Madrid, Spain (permits PROEX 128/15**)**. Thioglycolate-elicited peritoneal macrophages (PM) were isolated from 6-8-week-old pathogen-free mice. Cells were cultured in RPMI 1640 (2 mM L-glutamine, antibiotics 100 units/mL penicillin, 100 μg/mL streptomycin) with 5% FBS and seeded into 6-well-pates at a density of 1x10^6^ cells/well. Cells were allowed to adhere for 2 h and then the medium was changed to remove non-adherent cells. After 24 h, medium was replaced with new complete medium prior treatment.

### mRNA Isolation and RT-qPCR

Total cellular RNA was isolated using NZyol Reagent (NZYTech). cDNA was prepared by reverse transcription (GoTaq 2-Step RT-qPCR System, Promega) and amplified by PCR using SYBR^®^ Green PCR Master Mix and ABI Prism 7900HT sequence detection system (Applied Biosystems), with the following primers: mTNF-α F: CCACCACGCTCTTCTGTCTAC; R: AGGGTCTGGGCCATAGAACT; mIL-10 F: ATCGATTTCTCCCCTGTGAA; R: TGTCAAATTCATTCATGGCCT; mIL-12p40 F: TGGTTGCCATCGTTTTCCTG; R: ACAGGTGAGGTTCACTGTTTCT; mMKP-1 F: CTACCAGTACAAGAGCATCCC; R: AACTCAAAGGCCTCGTCCAG; mRPL13A F: ATCCCTCCACCCTATGACAA; R: GCCCCAGGTAAGCAAACTT. The 2−ΔΔCt method was applied to analyze the relative changes in expression profiling and all quantifications were normalized to the housekeeping gene RPL13A.

### ELISA

Cytokine concentration was determined for IL-10, TNF-α, IL2-p40 using DuoSet ELISA kit from R&D systems, according to the manufacturers protocol.

### Western Blot

Cells were lysed in ice-cold lysis buffer (50 mM Tris pH 7,5, 150 mM NaCl, 1% Triton X-100, 1 mM EDTA, 10% Glycerol), Phosphatase and Protease inhibitors (78440 Thermo Fisher). Equal protein amount (20 µg) from each cell lysate was separated on SDS 10% polyacrylamide gel and transferred to a nitrocellulose membrane (Bio-Rad). Membranes were blocked with 3% BSA for 1 h and incubated with the with antibodies against p-p38 (Thr180/Tyr182) (9211) (1:1000), total p38 (9212) (1:1000), p-ERK1/2 (Thr202/Tyr204) (9101) (1:1000), total ERK 1/2 (9102) (1:1000) from Cell Signaling, MKP-1 V-15 (sc-1199) (1:500) and β-actin (sc-47778) (1:2000) from Santa Cruz Biotechnology. The membranes were then incubated with the respective HPR-conjugated secondary antibody for 1 h and developed using ECL substrate (BioRad).

### Statistics

Analysis was performed using GraphPad Prism 5 software. Quantitative results are expressed as means ± SEM or mean ± SD. Statistical analysis was performed using one-way ANOVA followed by Bonferroni multiple comparisons. A p-value less than 0.05 was considered statistically significant

## Results

### TLR2 Ligands Induce Higher Levels of IL-10 than TLR4 Ligand LPS

**Figure 1:**
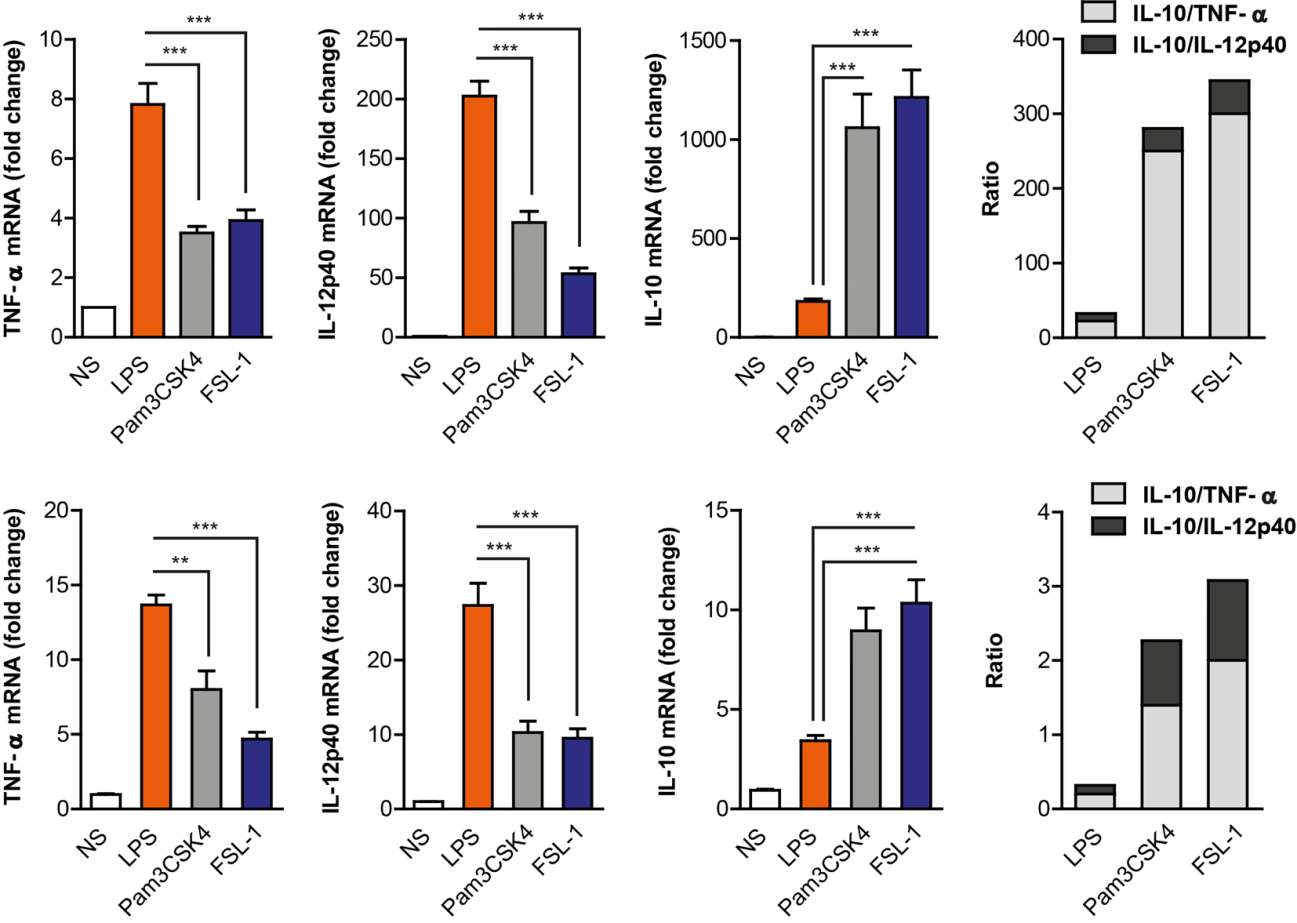
TLR4 and TLR2 ligands induce a distinct cytokine expression pattern in Raw264.7 cell line and peritoneal macrophages. Raw264.7 cells (upper graph) and mouse peritoneal macrophages (lower graph) were stimulated with LPS (100 ng/mL), Pam3CSK4 (1 µg/mL) and FSL-1 (100 ng/mL) for 24 h. Cytokine’s mRNA expression was assayed by quantitative RT-PCR, normalized to RPL13A, and presented relative to unstimulated cells (NS). Data shown is from a representative experiment of the three independent experiments performed from triplicate cultures (mean ± SEM) (**p < 0.01 and ***p < 0.001).

**Figure 2:**
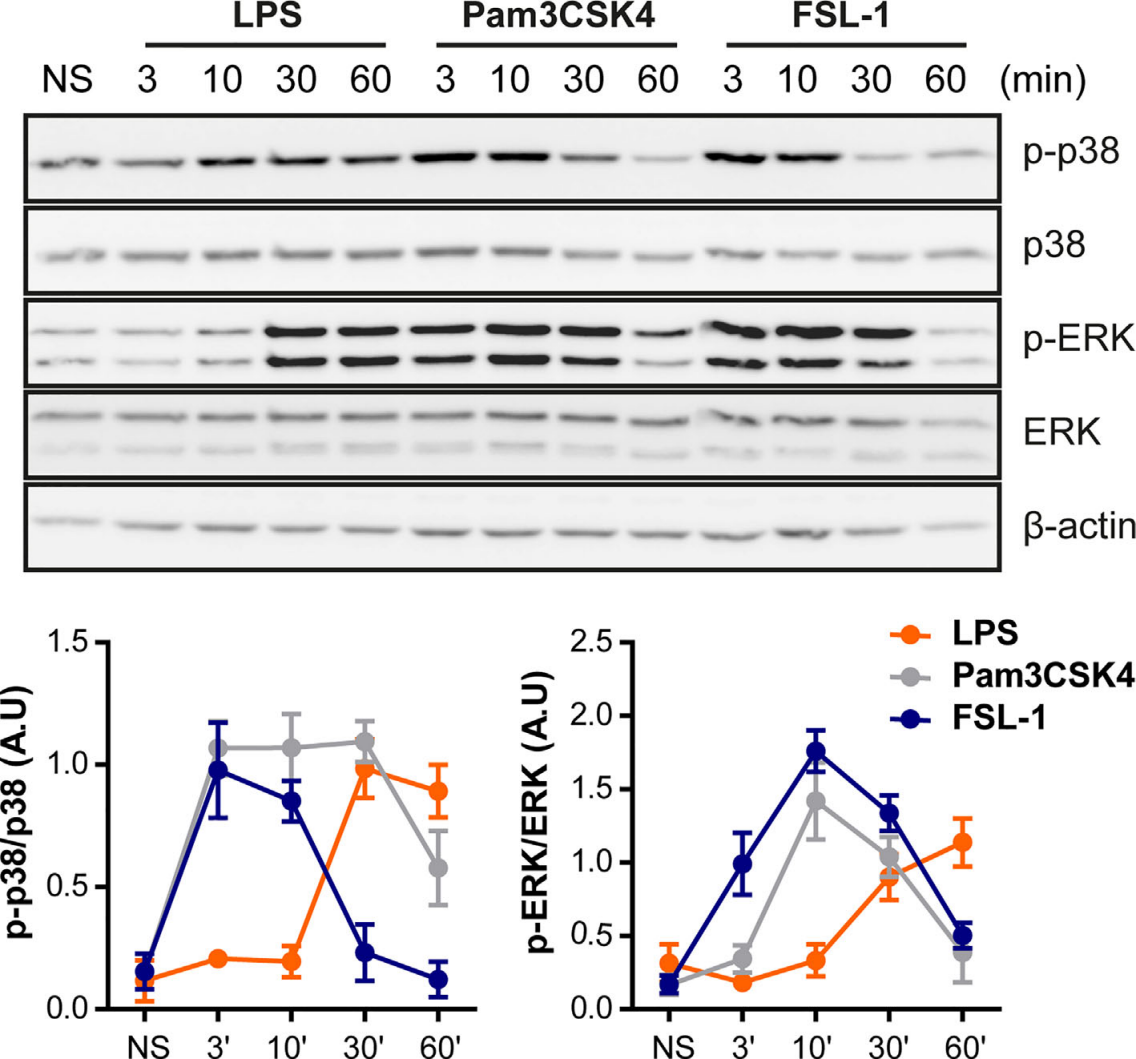
TLR4 and TLR2 ligands exhibit different kinetics on MAPK pathways activation. Raw264.7 cells were treated with LPS (100 ng/mL), Pam3CSK4 (1 µg/mL) and FSL-1 (100 ng/mL) at 3, 10, 30 and 60 min and stained with the antibodies indicated on the right side of the panels. The graphics below show the quantification of band intensities of the Western blots. The results are shown as mean ± SEM of three independent experiments.

TLR’s activation by various microbial-derived components induce distinct cytokine responses in macrophages that may fine-tune the acquired immune response. To determine whether TLR2 and TLR4 differ in their ability to activate macrophage responses, Raw264.7 cells were stimulated with specific ligands for TLR4 (LPS), TLR2/1 (Pam3CSK4) and TLR2/6 (FSL-1). The LPS stimulus induced higher transcription levels of IL-12p40 and TNF-α but lower induction of IL-10 cytokine than TLR2 ligands (**Figure 1**). This was observed over a wide range of ligand concentrations (**Supplementary Figure 1**). On the other hand, TLR2 ligands induced higher levels of IL-10, but lower activation of pro-inflammatory cytokines than LPS. Differences in IL-10 and IL-12 levels are more evident after 24 hours of stimulation which is the peak time for their secretion, as the opposite of TNF-α which is an earlier secreted cytokine (**Supplementary Figure 2**). To ensure that the differential cytokine induction observed in Raw264.7 cells was not a cell-line occurrence, the same experiment was performed using wild-type peritoneal macrophages (**Figure 2**). When these cells were treated with LPS, Pam3CSK4 or FSL-1, a similar pattern on cytokine’s induction was observed by TLR4 and TLR2 activation. This indicates that TLR2 ligands induce a more anti-inflammatory response compared to TLR4 ligands observed by the higher IL-10/IL-12p40 and IL-10/TNF-α ratios. These results demonstrate that both macrophage cell types (cell line vs primary cell) have a similar cytokine induction profile *via* TLR4 and TLR2 receptors.

### TLR2 Activation Induces p38 and ERK Phosphorylation Earlier and Faster Compared to TLR4

Since TLR4 and TLR2 receptor’s activation induce a distinct cytokine pattern, the next step was to examine whether TLR4 and TLR2 ligands could differentially activate some of the cell signaling pathways. Raw264.7 cells were serum starved and then stimulated for 3, 10, 30 and 60 minutes with LPS, Pam3CSK4 or FSL-1. We detected MAPKs ERK and p38 activation in response to LPS, observing phosphorylation around 30 and 60 min (**Figure 2**). Interestingly, TLR2 ligands activate MAPK signaling pathways faster; by 3 min after stimulation, phospho-ERK and phospho-p38 bands were clearly visible. Furthermore, p38 and ERK phosphorylation induced by TLR2 declines faster, at 30 and 60 min compared with the TLR4 ligand LPS, which still maintains at 60 min. For longer time points p38 shows more activation phases (phosphorylation band at 2,15, and 24 h) similar for LPS, Pam3CSK4 and FSL-1 stimulation (**Supplementary Figure 3**). This indicates a differential p38 activation by TLR4 and TLR2 ligands in the first 60 min of stimulation. In peritoneal macrophages, a similar early kinetic signature was observed (**Supplementary Figure 4**). Peritoneal macrophages from TLR2 KO and TLR4 KO mice were also used to confirm the ligands stimulation-specificity. Upon stimulation of TLR2 KO macrophages, TLR2 ligands were unable to activate MAPK pathways, indicating the specificity of these ligands for TLR2 receptor. As expected, LPS was able to activate these cells but not TLR4 KO macrophages

**Figure 3:**
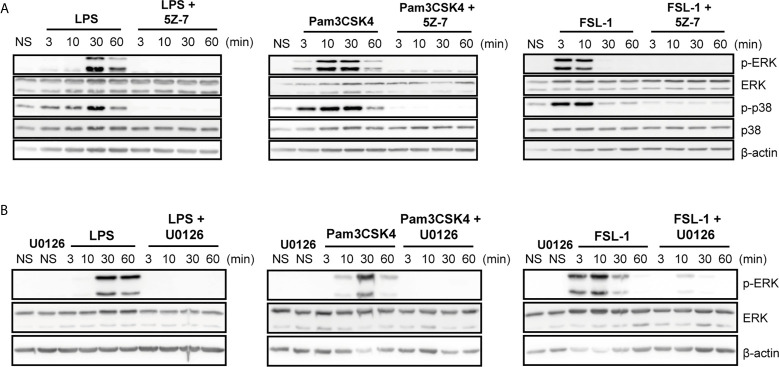
TAK1 and MEK1/2 are upstream activators of ERK and p38 regardless of the TLR stimuli in peritoneal macrophages. Peritoneal macrophages were pretreated with 1 µM 5Z-7-oxzeaenol (“5Z-7”) **(A)** or with 1 µM U0126 **(B)** for 30 min. Cells were left unstimulated (NS) or stimulated with LPS (100 ng/mL), Pam3CSK4 (1 µg/mL) and FSL-1 (100 ng/mL) at 3, 10, 30 and 60 min. A representative western blot is shown of three independent experiments.

To study the differential kinetic signaling between TLR4 and TLR2 ligands, special focus was given to investigate whether different upstream ERK and p38 activators could be induced *via* TLR4 or TLR2 activation. To this end, macrophages were pretreated with TAK1 inhibitor (5Z-7-oxzeaenol) and MEK 1/2 inhibitor (U0126) once these mediators are described to be implicated in ERK activation. In the presence of TAK1 inhibitor, ERK and p38 phosphorylation were strongly reduced upon stimulation with TLR4 and TLR2 ligands, both in peritoneal macrophages (**Figure 3A**) and Raw264.7 cells (**Supplementary Figure 5A**) suggesting that TAK1 is an upstream activator for these MAPKs. Similarly, the inhibition of MEK by U0126 produced similar results with all ligands in Peritoneal (**Figure 3B**) and Raw264.7 cells (**Supplementary Figure 5B**)

### MKP-1 Is Activated Earlier by TLR2 Activation and Controls TLR2-Induced p38 Phosphorylation

**Figure 4:**
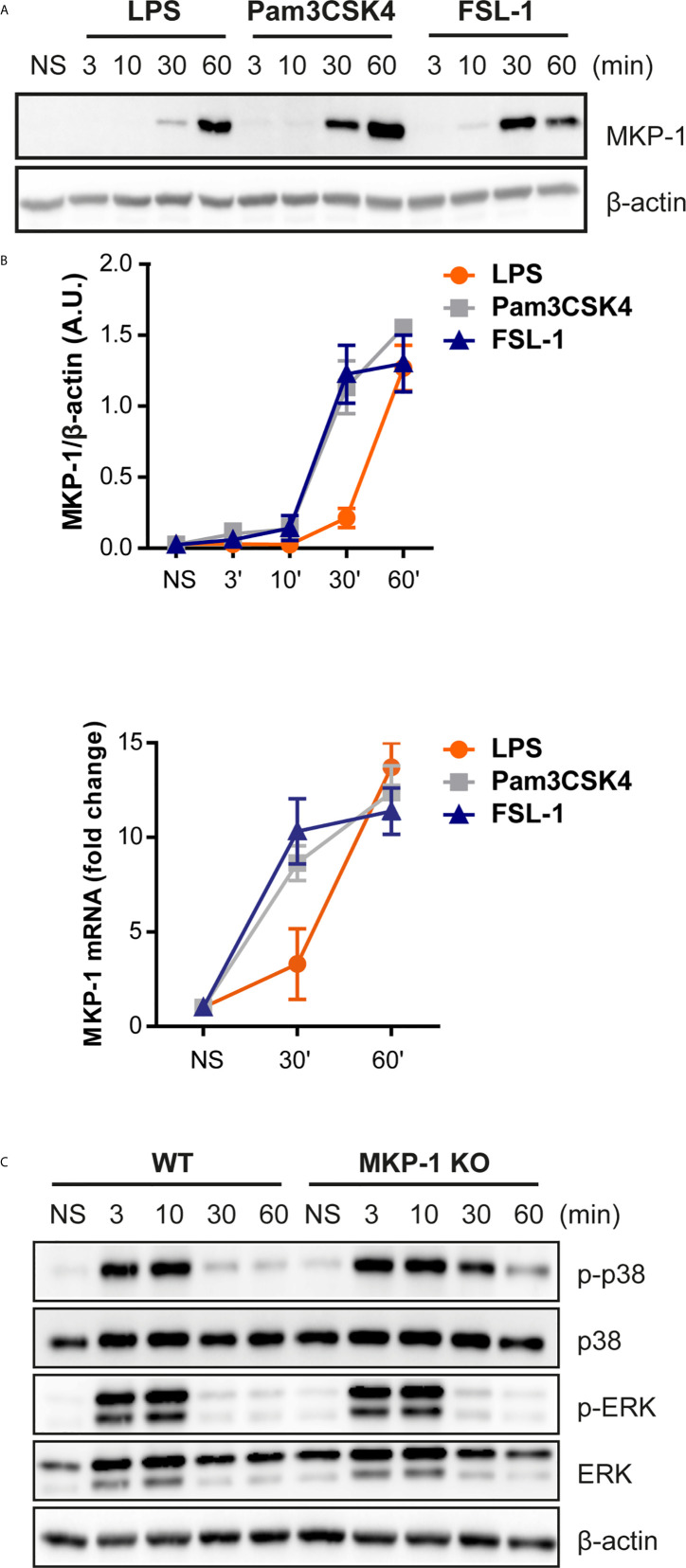
MKP-1 is activated earlier by TLR2 activation and controls TLR2-induced p38 phosphorylation. **(A)** Raw264.7 cells were left unstimulated (NS) or stimulated with LPS (100 ng/mL), Pam3CSK4 (1 µg/mL) and FSL-1 (100 ng/mL) at 3, 10, 30 and 60 min and protein expression was measured by Western blot. **(B)** MKP-1 mRNA levels were measured by qPCR after Raw 264.7 cells stimulation with the three ligands at 30 and 60 min. **(C)** WT and MKP-1 KO peritoneal macrophages were left unstimulated (NS) or stimulated FSL-1 (100 ng/mL) at 3, 10, 30 and 60 min. β-actin was used as protein loading control. Images are representative of two experiments.

Since no clear effect in upstream kinase(s), and to further understand the cause of differential kinetics on p38 and ERK rapid phosphorylation by TLR2 activation, we addressed the possible role of phosphatases, more specifically MKP-1. This phosphatase was described to be induced and stabilized through phosphorylation by ERK in LPS-stimulated cells. Moreover, MKP-1 has higher affinity to dephosphorylate p38 and JNK rather than ERK (14). Stimulation of Raw264.7 cells and peritoneal macrophages with the TLR ligands exhibit MKP-1 induction (**Figure 4A**). In response to LPS, MKP-1 was induced at 60 min after stimulation, whereas in response to TLR2 ligands MKP-1 was detected earlier, at 30 min. A similar kinetic pattern was observed for MKP-1 transcription at 30 and 60 min (**Figure 4B**), however in peritoneal macrophages MKP-1 mRNA levels started to decay at 60 min (**Supplementary Figure 6**). This data indicates that TLR2 ligands induce MKP-1 transcription and expression earlier compared to the TLR4 ligand LPS.

Previous data demonstrated that MKP-1 controls p38 activation in response to TLR4 activation (13, 15). Therefore, the MKP-1 role in MAPKs activation in response to FSL-1 was addressed. WT and MKP1-deficient macrophages were treated with FSL-1 for 60 min. Macrophages deficient in MKP-1 exhibited a prolongation of p38 phosphorylation but not for ERK comparing with WT (**Figure 4C**). Therefore, besides TLR4, MKP-1 will preferably regulate p38 phosphorylation in response to TLR2/6 stimulation.

### Early p38 Activation Is Determinant for IL-10 Expression by TLR2 Stimulation

**Figure 5:**
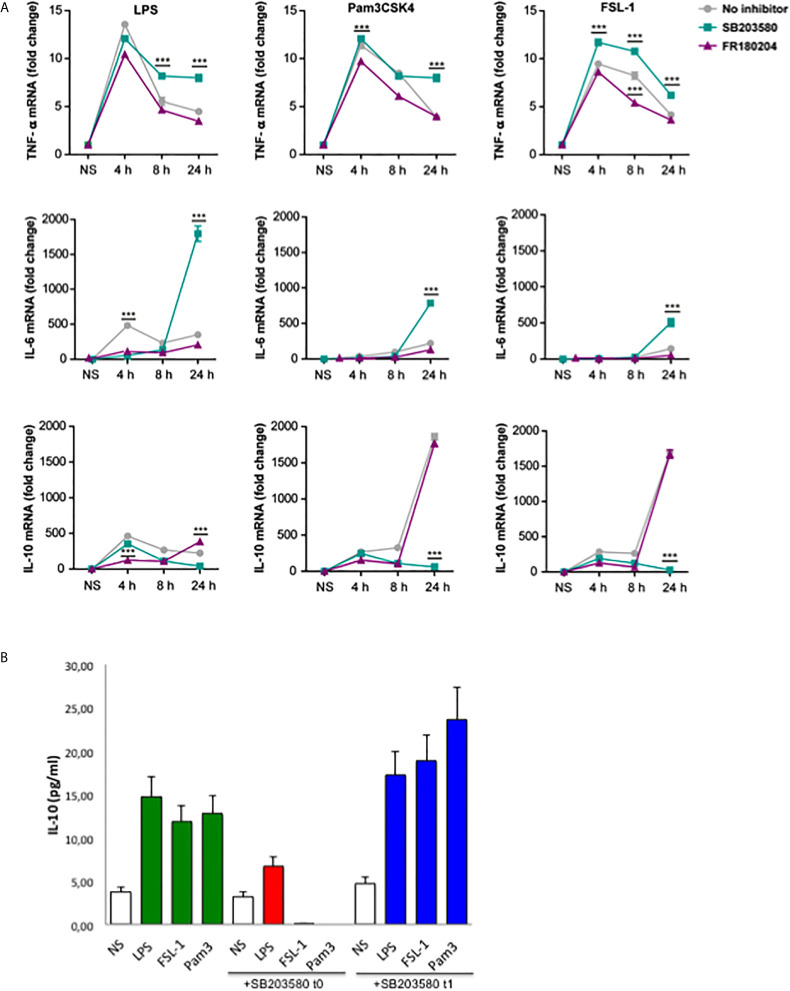
Early p38 activation contributes for later IL-10 production in response to TLR2 ligands. **(A)** Raw264.7 cells were pre-treated with 10 µM SB203580 or 10 µM of FR180204 for 30 min and then stimulated with LPS (100 ng/mL), Pam3CSK4 (1 µg/mL) and FSL-1 (100 ng/mL) for 24 hours. Cytokines mRNA expression was assayed by quantitative RT-PCR, normalized to RPL13A, and presented relative to unstimulated cells (NS). Data from triplicate cultures are representative of three independent experiments (mean ± SEM.) (***p < 0.001). **(B)** Raw264.7 cells were treated with 10 µM SB203580 at the same time (t0) or 60 min (t1), after stimulation with LPS (100 ng/mL), Pam3CSK4 (1 µg/mL) and FSL-1 (100 ng/mL). Cell supernatants were collected after 24 hour-stimulation and IL-10 cytokine protein levels were measured by ELISA. Data are the mean of 2 experiments.

Published data describes that IL-10 expression is dependent of p38 activation in response to LPS (12, 13, 16). Thus, the next step was to investigate the effect of p38 and ERK inhibition in the cytokine’s induction by the TLR2 ligands. A time-course kinetics was performed to determine the changes of cytokine levels in Raw264.7 cells pretreated with p38 and ERK inhibitors, SB203580 or FR180204 respectively, and stimulated with TLR4 or TLR2 ligands for 4, 8 and 24 hours (**Figure 5A**). Stimulation with all TLR ligands gave a similar pattern for TNF-α induction. However IL-6 levels by TLR2 ligands were lower at earlier time points compared to the levels induced by LPS At 24 hours, p38 inhibition resulted in TNF-α and IL-6 induction in response to Pam3CSK4 and FSL-1, similar to the effect observed in response to LPS. More importantly, IL-10 levels are highly increased at 24 hours in response to TLR2 ligands compared to TLR4, being this effect fully dependent of p38. Inhibition of ERK produced variable effect depending on the time point tested. To confirm that early p38 activation by TLR2 ligands is decisive for the high levels of IL-10 expression, Raw264.7 cells were treated with p38 inhibitor 60 min after TLR stimulation with LPS, Pam3CSK4 or FSL-1 (**Figure 5B**). The inhibitor addition 1h post TLR stimulation does not impair IL-10 levels, indicating that the early p38 activation is determinant for the high IL-10 expression levels observed for TLR2 stimulation.

**Figure 6:**
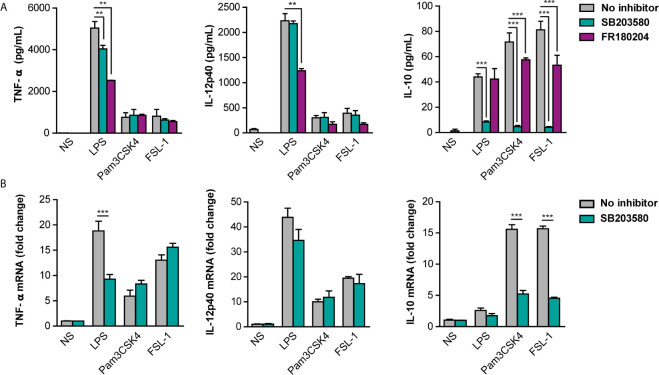
Differential contribution of p38 and ERK for cytokine production in response to TLR ligands. Peritoneal macrophages were pre-treated with 10 µM SB203580 or 10 µM FR180204 for 30 min and then stimulated with LPS (100 ng/mL), Pam3CSK4 (1 µg/mL) and FSL-1 (100 ng/mL) for 24 hours. **(A)** Cytokine protein levels and **(B)** Cytokine mRNA levels were measured by ELISA and qPCR respectively. Data from triplicate cultures (mean ± SEM.) are representative of one of the three independent experiments performed (**p < 0.01 and ***p < 0.001).

The effect of p38 and ERK inhibition on cytokine’s expression was further evaluated in peritoneal macrophages pretreated with p38 and ERK inhibitors and stimulated with TLR ligands for 24 hours. Interestingly, IL-12 was not affected in the presence of the p38 inhibitor in response to all TLR ligands and pro-inflammatory TNF-α only weakly in response to LPS (**Figure 6A**). On the other hand, IL-10 levels were strongly decreased by p38 inhibition. A similar pattern was observed for the cytokine transcription levels in stimulated-peritoneal macrophages (**Figure 6B**). ERK inhibition by FR180204 had no effect on LPS-induced IL-10 although decreased slightly the production of this cytokine in response to TLR2 ligands (**Figure 6A**). Nonetheless, ERK inhibition affected TNF-α and IL-12 production by LPS. These results imply that ERK and p38 are important for pro-inflammatory cytokines induction at early time points of inflammation, whereas p38 but not ERK contributes for later anti-inflammatory IL-10 production in response to TLR4 and TLR2 ligands.

## Discussion

Our results show that stimulation of macrophages with TLR4 or TLR2 ligands resulted in striking differences in cytokine production. Specifically, TLR2/6 and TLR2/1 ligands exhibit a higher induction of the anti-inflammatory mediator IL-10, whereas TLR4 favors the expression of pro-inflammatory or Th1 cytokines such as TNF-α and IL-12p40. The higher IL-10/TNF-α and IL-10/IL-12p40 ratios observed in macrophages activated by TLR2 ligands suggest a change to a M2 and regulatory phenotype, whereas LPS induces an M1-type macrophage as previously described. This is concordant with previous published data in human DCs stimulated with LPS, PGN, zymosan and Pam3Cys, where TLR2 ligands favored a Th2 response in contrast to LPS (17–19). In this regard, several pathogens, such as *Brucella abortus*, *Candida albicans* or *Mycobacterium tuberculosis* may explore TLR2-mediated IL-10 induction as a virulence mechanism to induce M2 macrophages and suppress Th1 response against the pathogen (20, 21). Given this distinct cytokine profile, it was questionable if TLR4 and TLR2 ligands could activate exactly similar signaling pathways, including the activation MAPKs p38 and ERK. Our results demonstrate different kinetics of activation between TLR4 and TLR2 ligands, however Pam3CSK4 and FSL-1 show similar kinetics of activation, which is in accordance with previous studies (22). Our experiments were performed in both a macrophage cell line and primary macrophages, thus showing that this differential kinetics is an important property of TLR signaling in macrophages.

A possible explanation for the differential MAPKs activation could be that TLR4 and TLR2 ligands would activate different upstream mediators of p38 and ERK. TAK1 is a general upstream kinase described to be important for activation of NF-κB, p38, JNK and ERK (23) Our results show that TLR4 and TLR2 ligands are dependent of TAK1 to induce ERK and p38 and this is observed for the Raw264.7 cell line as well as for peritoneal macrophages. Moreover, our results with U0126-specific MEK1/2 is inhibitor also suggested that no major differences in the activation of this pathway by TLR2 an TLR ligands may be responsible for differential ERK activation. Many pathways could be potential mediators of this differential early signaling between TLR2 and TLR4 ligands, including TPL2, Ras, PKC zeta, PI3K, etc., that deserves further research.

On the other hand, dephosphorylation of MAPKs is one of the most efficient mechanisms to control their activation. Dual specificity phosphatases (DUSP), also termed MKP can negatively regulate MAPKs activation through dephosphorylation of phosphotyrosine and phosphothreonine residues (24). DUSPs can be induced by TLR-mediated activation of MAPKs, creating a negative feedback loop to limit MAPKs activation. Among DUSPs, MKP-1 is highly expressed in macrophages (25). Our results show that both TLR4 and TLR2 ligands induce MKP-1, however with different kinetics of activation, similarly to ERK and p38 MAPKs. TLR2/1 and TLR2/6 induced a faster activation of the MKP1 phosphatase than TLR4 coincident with the sudden drop in phosphorylated ERK. Thus, it is likely that this temporal difference in MKP-1 induction *via* TLR4 and TLR2 may be responsible for the same differences observed in ERK and p38 phosphorylation.

MKP-1 induction is determinant for controlling the length of MAPK responses in macrophages. Previous studies demonstrated that MKP-1 KO peritoneal, alveolar macrophages and bone marrow-derived macrophages showed prolonged activation of p38 and JNK, suggesting that MKP-1 regulates primarily p38 and JNK, but has little effect on ERK, in response to LPS (13, 26, 27). Our results show a similar result in response to the TLR2 ligand FSL-1, where MKP-1 KO PM exhibit a prolonged p38 phosphorylation whereas ERK phosphorylation was not affected. In the absence of MKP-1, p38 is likely to eventually become inactivated by other MKPs, though at a much slower rate. This is consistent with the observation that deactivation of p38 in MKP-1 KO macrophages was delayed. Overall, MKP-1 has a central role in the regulation of p38 by TLR2 signaling in macrophages.

Importantly, our results also demonstrate that p38 is critical for later anti-inflammatory IL-10 induction but not for pro-inflammatory or Th1 cytokines, regardless of the stimuli. This is in accordance with Chi et al, which demonstrates that IL-10 induction mediated by LPS was blocked by a p38 inhibitor, implying that p38 mediates IL-10 synthesis (13). Our results indicate that TLR2 ligands also induce IL-10 expression fully dependent of p38 at later stages. Importantly, the higher levels of IL-10 expression induced by TLR2 ligands were proved to be dependent on early activation of p38, in contrast with the later activation by LPS. The kinetics of p38 blockade indicates that this window period 0-1 h is crucial for sequential IL10 production. Furthermore, it is described that IL-10 inhibits the transcription of pro-inflammatory cytokines *via* IL10R-Janus kinase-STAT3 pathway (28). Therefore, the earlier activation of this pathway could contribute for the higher IL-10 levels and lower pro-inflammatory cytokines observed *via* TLR2 activation. Transcription factors also influence cytokine expression. For example, SP1, c/EBPβ, CREB/AP1 and MAF have been shown to bind to IL-10 promoter. MSK1/MSK2-mediated activation of CREB and ATF1 induced IL-10 in response to LPS (29). However, the different stimuli may induce different transcription factors activated in a same cell type. For example, p38 induce IL-10 promoter *via* SP1 in response to LPS, whereas c/EBP5 was found to be recruited in response to cAMP (12, 30). Further studies are required to identify the downstream targets activated by early p38 phosphorylation that mediate IL-10 regulation in response to TLR2 ligands.

In addition, ERK activation seems to mediate some of the responses of TLR4, mostly pro-inflammatory or Th1 cytokines, since ERK inhibitors prevent partially LPS response. However, ERK activation did not affect on TLR2/1 and TLR2/6 mediated pro-inflammatory or Th1 cytokines and slightly IL-10 cytokine induction. This point out to a different involvement of MAPK, ERK and p38 in cytokine regulation depending on the nature of the foreign invader pathogen sensed by different TLRs.

**Figure 7:**
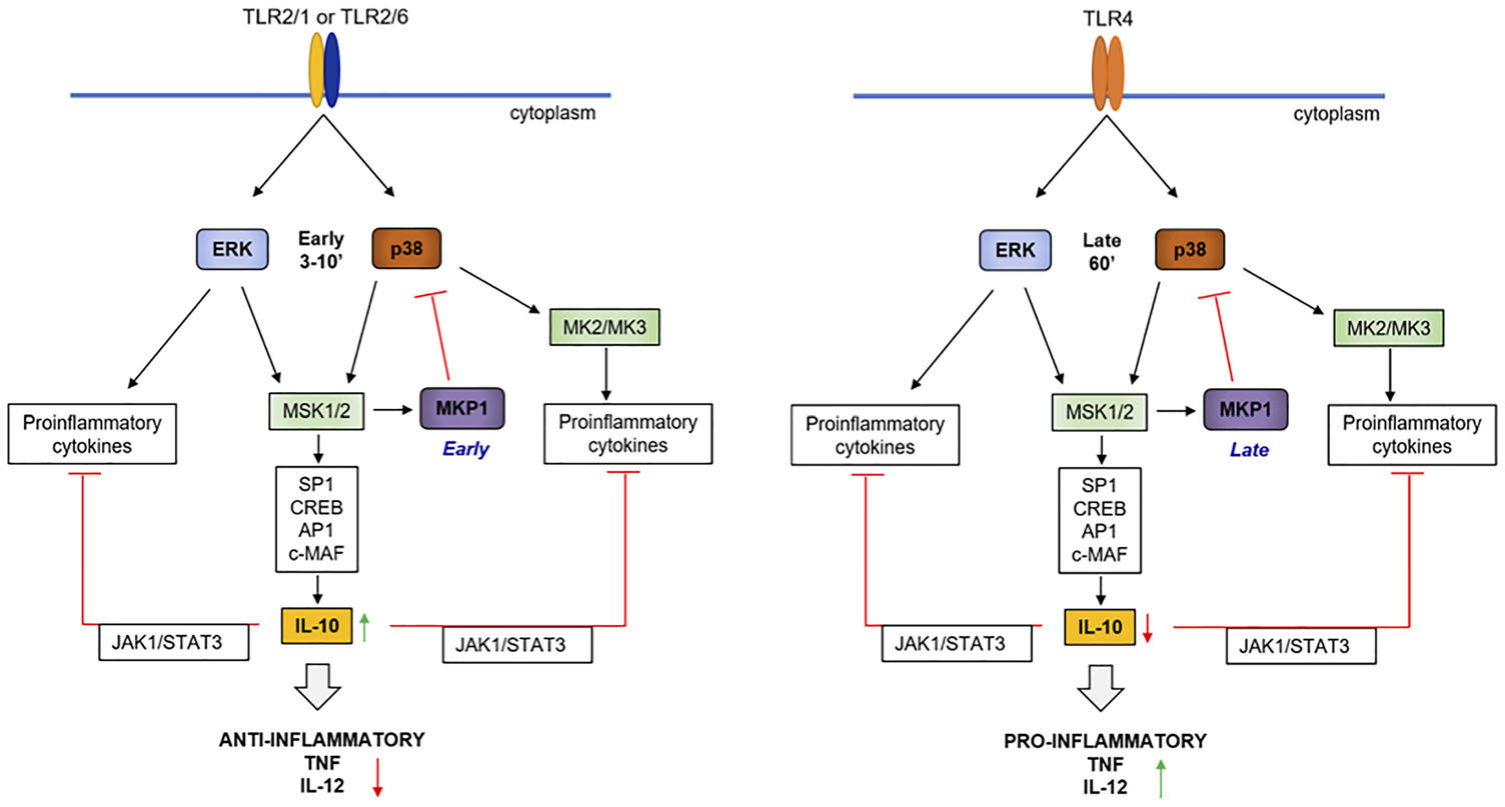
Hypothetical model of the molecular events leading to high IL-10 levels in response to TLR2 ligands. TLR2 ligands such as Pam3CSK4 or FSL-1 activate NF-κB, ERK and p38 earlier and faster (≈ 3 min) compared to LPS (≈ 30-60 min), allowing a faster induction of cytokines such as TNF-α, IL-12, IL-6 and IL-10 by ERK. The earlier induction of MKP-1 by TLR2 activation contributes for upregulation of pro-inflammatory cytokines and MKP-1 can dephosphorylate p38. On the other hand, the early TLR2-mediated p38 induction contributes for later and high IL-10 levels of expression through activation of transcription factors such as SP1, CREB, AP1 and c-MAF. Consequently, IL-10 activates the STAT3 pathway, which amplifies IL-10 production and regulates pro-inflammatory cytokine induction.

In summary, our results indicate that TLR2 ligands induce higher levels of anti-inflammatory IL-10 and lower levels of pro-inflammatory cytokines IL-12, TNF-α compared to TLR4 ligands in macrophages. Our results support a model (**Figure 7**) in which TLR2 activates MAPKs faster and MKP-1 controls p38 phosphorylation which differentially controls pro and anti-inflammatory cytokines. Our work shows for the first time that early and fast p38 activation in response to TLR2 ligands is critical for the higher levels of IL-10 induction compared to TLR4, leading to reduced pro-inflammatory cytokines.

## Data Availability Statement

The original contributions presented in the study are included in the article/**Supplementary Material**. Further inquiries can be directed to the corresponding author.

## Ethics Statement

The animal study was reviewed and approved by Comité de Ética de la Dirección General del Medio Ambiente de la Comunidad de Madrid, Spain (permits PROEX 128/15).

## Author Contributions

SF, AA, and MF designed the experiments. SF, CP, and AA performed the experiments. RP helped with macrophage KO mice experiments. SF and MF wrote the manuscript. All authors contributed to the article and approved the submitted version.

## Funding

This research was funded by grants from European Unión “Host-microbe interactions in Health and disease. Interface with the immune system”. HOMIN - 317057 - FP7-PEOPLE-2012-ITN to Diomune and Ministerio de Ciencia e Innovación (SAF2013-42850-R and SAF2016-75988-R), Instituto de salud Carlos III “ (BIOIMID and RD16/0027/0006), Comunidad de Madrid (S2017/BMD-3671. INFLAMUNE-CM) to MF, and Institutional grants from “Fundación Ramón Areces” and “Banco de Santander”.

## Conflict of Interest

The authors declare that the research was conducted in the absence of any commercial or financial relationships that could be construed as a potential conflict of interest.
