# Supplementary material for: Early p38 Activation Regulated by MKP-1 Is Determinant for High Levels of IL-10 Expression Through TLR2 Activation
Source: Front Immunol. 2021 Jun 21;12:660065. doi: 10.3389/fimmu.2021.660065 (PMC8256158; doi:10.3389/fimmu.2021.660065)
Supplement: Supplementary file 1 [file DataSheet_1.docx]

**Supplementary information**

**Title:** Early p38 activation regulated by MKP-1 is determinant for high levels of IL-10 expression through TLR2 activation

**Authors:** Sara Francisco, Alicia Arranz, Javier Merino, Carmen Punzón, Rosario Perona, and Manuel Fresno

**Supplementary Figure 1**

**
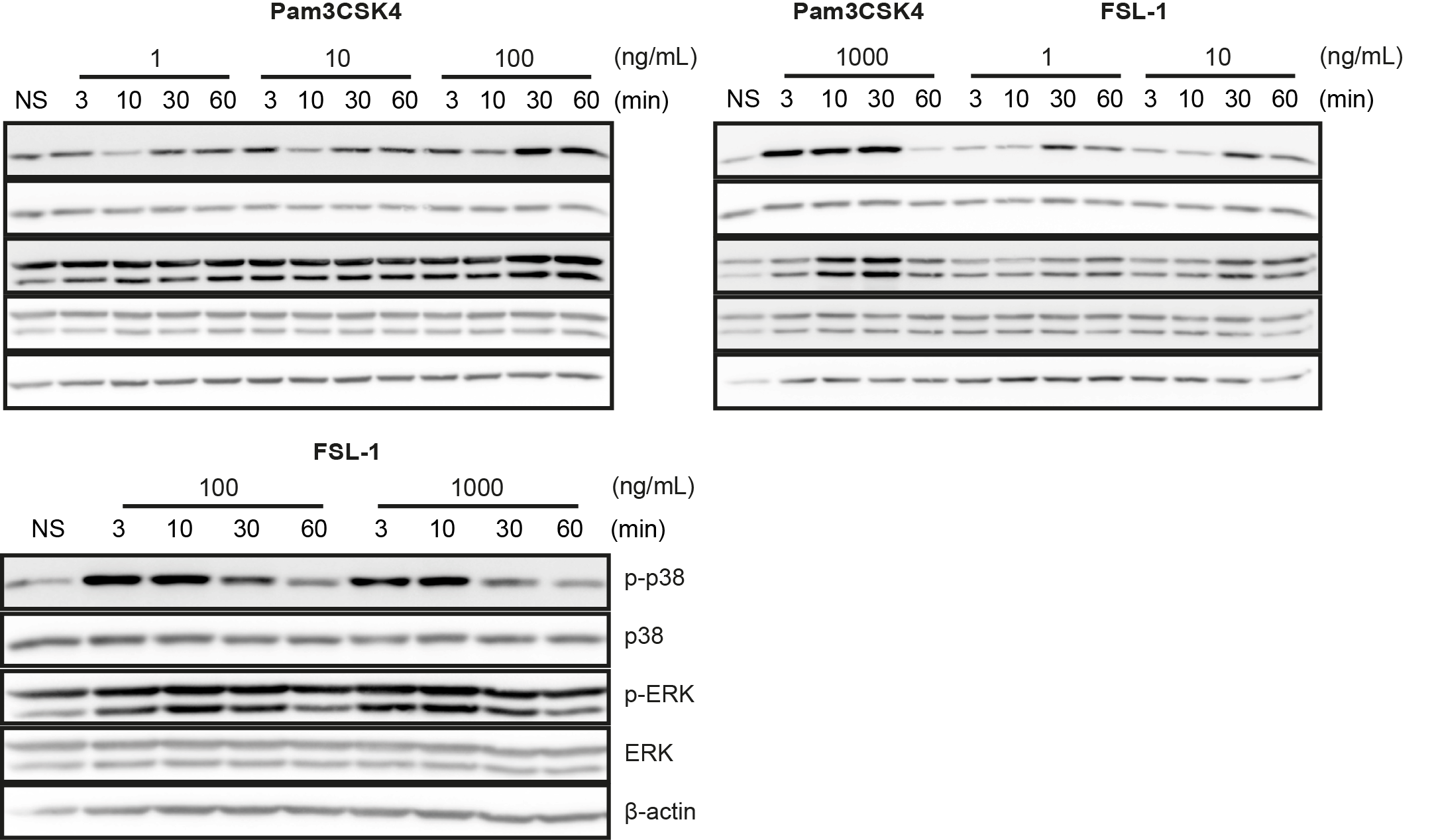
**

**Supplementary Fig. S1 Early p38 and ERK phosphorylation by TLR2 activation is ligand-dose dependent.**

Raw264.7 cells were stimulated with incremental doses of Pam3CSK4 or FSL-1 (1,10,100 and 1000 ng/ml), indicated in the image at 3, 10, 30 and 60 min and stained with the indicated antibodies. The image is representative of one experiment.

**
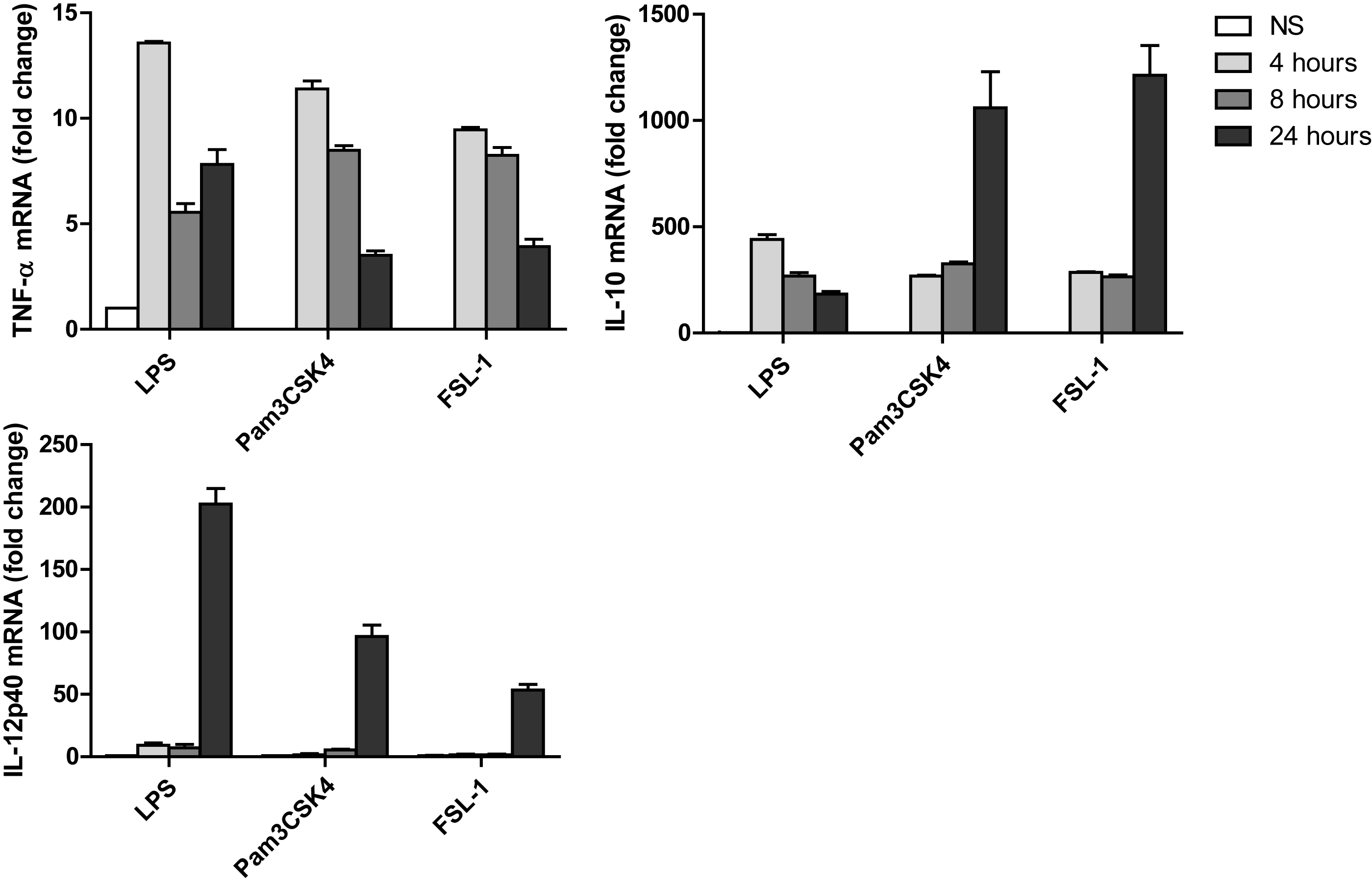
Supplementary Figure 2Supplementary Fig. S2 Cytokine expression upon TLR4 and TLR2 activation in Raw264.7 cell line.**

Raw264.7 cells were stimulated with LPS (100 ng/mL), Pam3CSK4 (1 µg/mL) and FSL-1 (100 ng/mL) for 4, 8 and 24 h. Cytokine’s mRNA expression was assayed by quantitative RT-PCR, normalized to RPL13A, and presented relative to unstimulated cells (NS). Data are representative of two independent experiments.

**
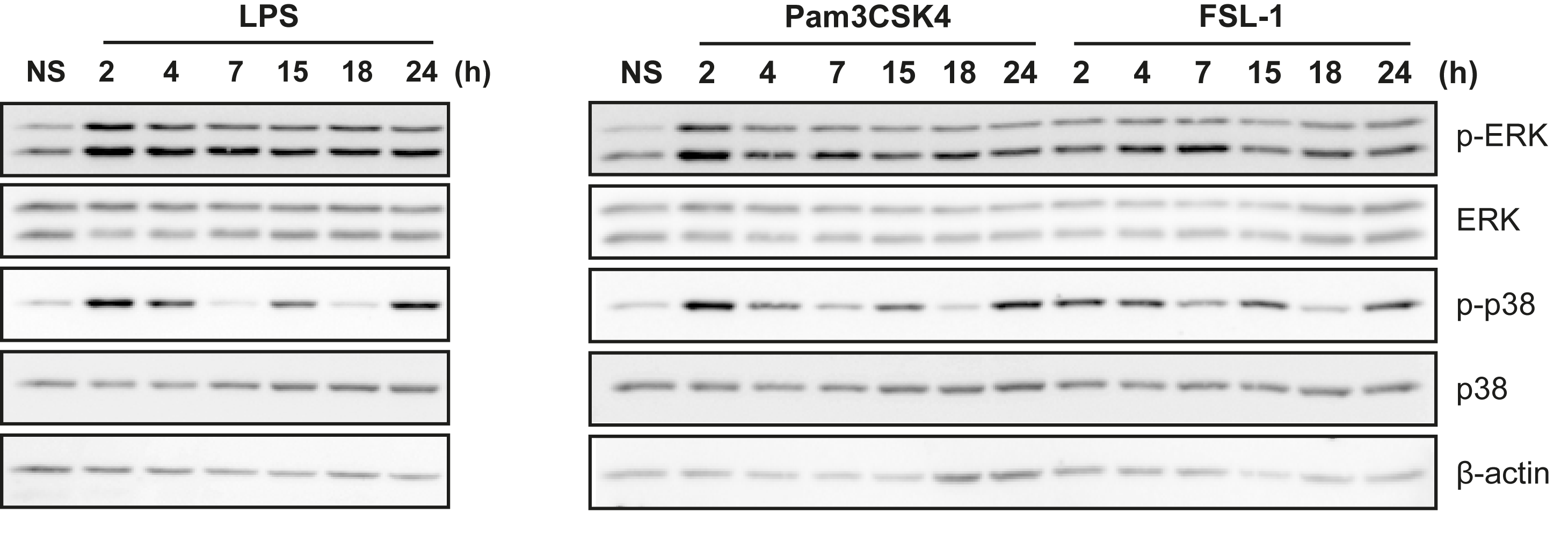
Supplementary Figure 3**

**Supplementary Fig. S3 p38 and ERK activation kinetics upon stimulation by LPS, Pam3CSK4 and FSL-1 in Raw264.7 cell line.**

Raw264.7 cells were treated with LPS (100 ng/mL), Pam3CSK4 (1 µg/mL) and FSL-1 (100 ng/mL) at 2, 4, 7, 15, 18 and 24 hours and stained with the antibodies indicated on the right side of the panels. The image is representative of one experiment.

**Supplementary Figure 4**


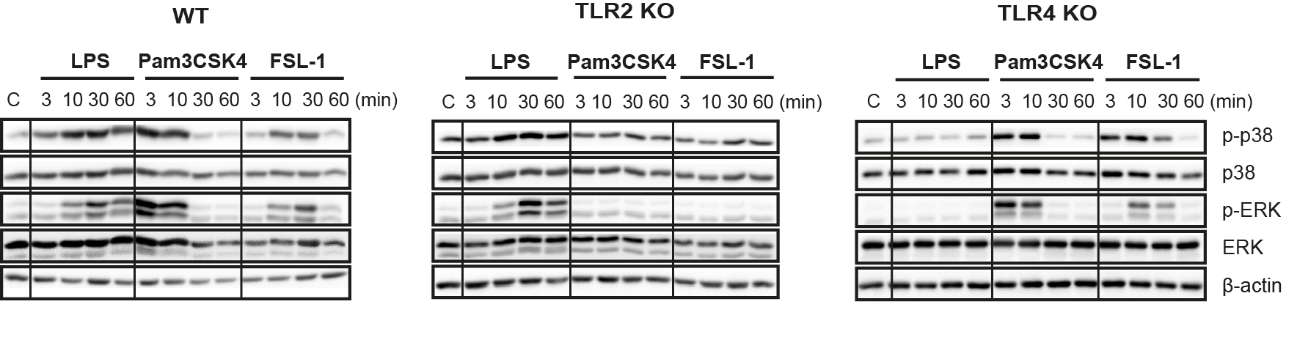


**Supplementary Fig. S4 TLR4 and TLR2 ligands responses are specific of TLR4 and TLR2 receptor, respectively.**

WT peritoneal macrophages, TLR2 KO and TLR4 KO peritoneal macrophages were left untreated (c) or treated with LPS (100 ng/mL), Pam3CSK4 (1 µg/mL) and FSL-1 (100 ng/mL) at 3, 10, 30 and 60 min. The data are representative of two independent experiments.

**
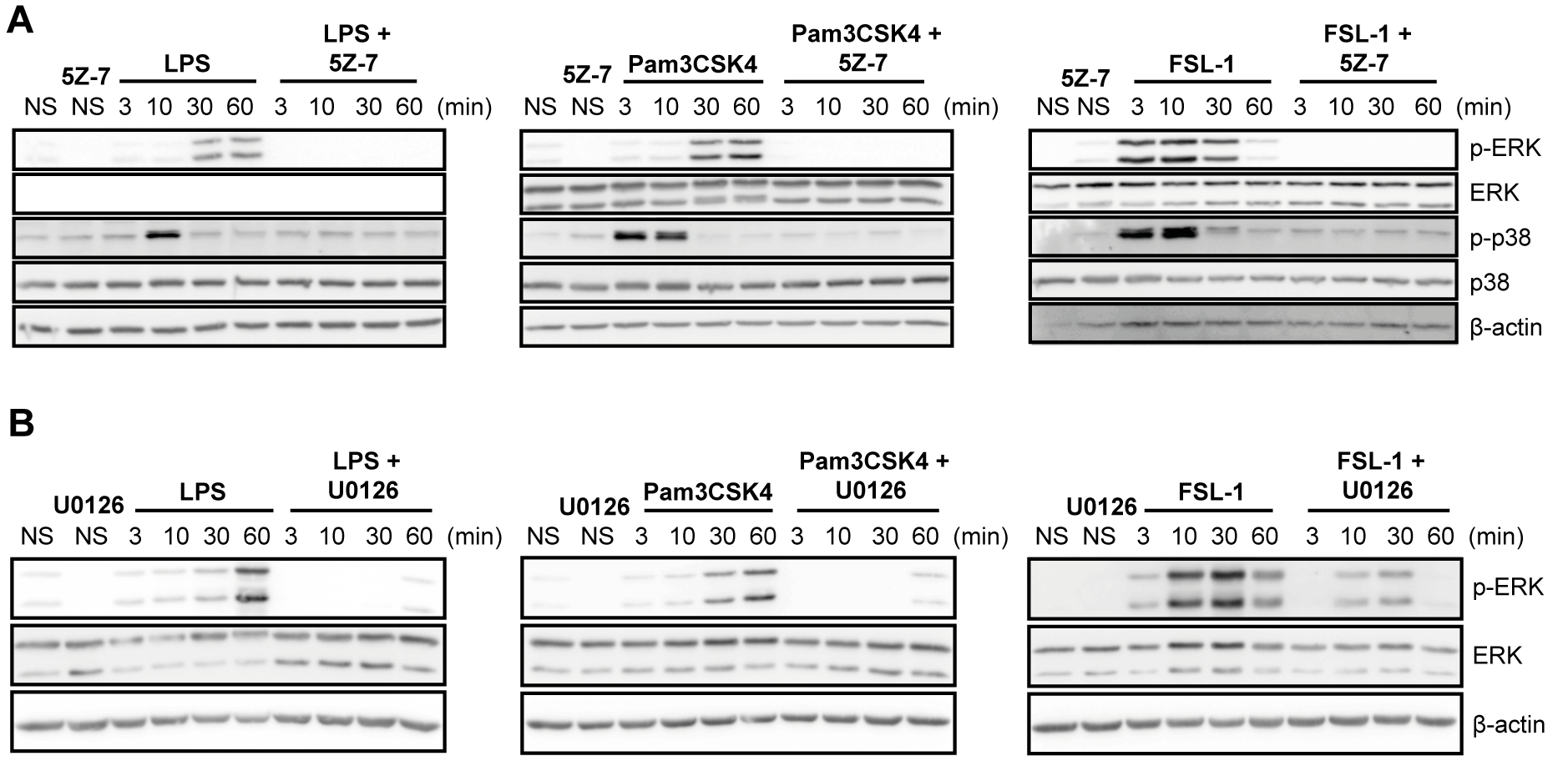
Supplementary Figure 5**

**Supplementary Fig. S5** **TAK1 and MEK1/2 are upstream activators of ERK and p38 regardless of the TLR stimuli in Raw 264.7 cell line.**

Cells were pretreated d with 1 µM 5Z-7-oxzeaenol (“5Z-7”) (A) or with 1 µM U0126 (B) for 30 min. Cells were left unstimulated **(NS)** or stimulated with LPS (100 ng/mL), Pam3CSK4 (1 µg/mL) and FSL-1 (100 ng/mL) at 3, 10, 30 and 60 min. A representative western blot is shown of three independent experiments.

**Supplementary Figure 6**


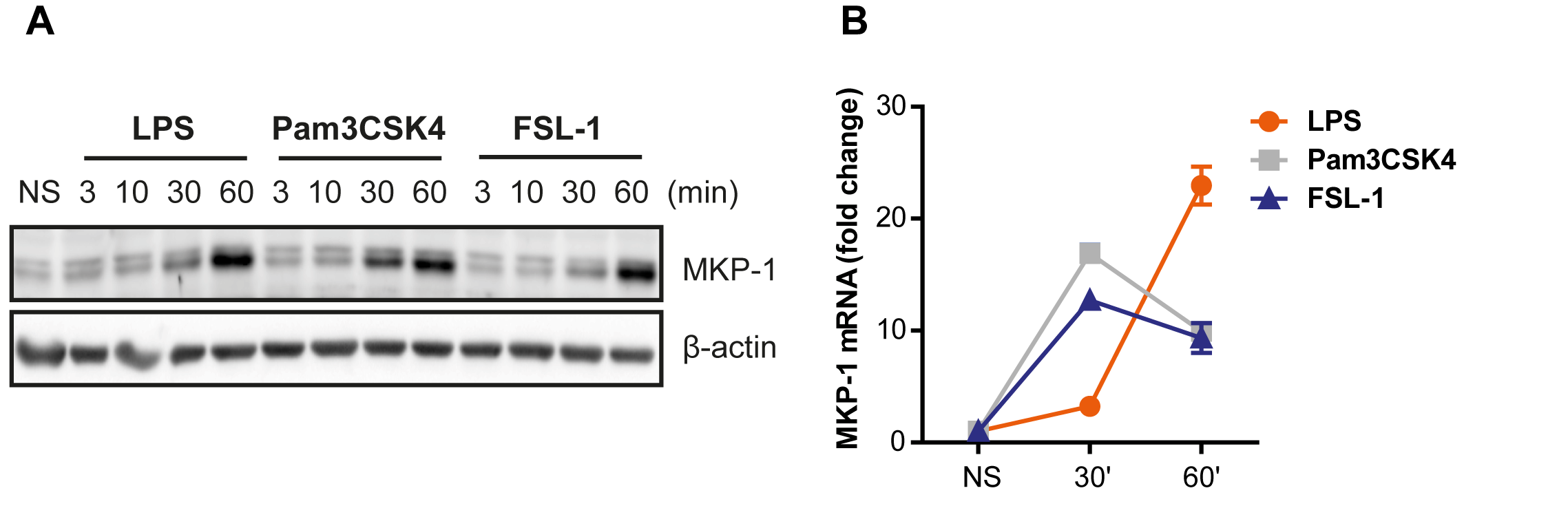


**Supplementary Fig. S6 MKP-1 is activated earlier by TLR2 activation in peritoneal macrophages.**

A) Mouse peritoneal macrophages were left unstimulated (NS) or stimulated with LPS (100 ng/mL), Pam3CSK4 (1 µg/mL) and FSL-1 (100 ng/mL) at 3, 10, 30 and 60 min and protein expression was measured by Western blot. B) MKP-1 mRNA levels after cell stimulation with the three ligands at 30 and 60 min were measured by qPCR. The data are representative of two independent experiments.
